# Body Image, Self-Esteem, and Attitudes Toward Sexuality in Older Adults with Chronic Illnesses

**DOI:** 10.1007/s10508-025-03146-x

**Published:** 2025-04-28

**Authors:** Hediye Özbay, Adil Utli, Nilay Filoğlu Ersü

**Affiliations:** 1 Department of Elderly Care, Vocational School of Health Services, Mardin Artuklu University, Artuklu Campus, 47200 Mardin, Turkey; 2 Faculty of Medicine, Department of Emergency Medicine, Ege University, İzmir, Turkey; 3 Department of Midwifery, Mardin Artuklu University, Mardin, Turkey

**Keywords:** Body image, Chronic illness, Elderly, Self-esteem, Sexuality

## Abstract

This study aimed to examine the effect of the association between body image and self-esteem levels on attitudes toward sexuality in older adults with chronic illnesses. The research was descriptive, cross-sectional, and correlational. The study sample comprised 1,004 people over the age of 65 who visited family health centers in a province in the east of Türkiye. The Older Person’s Description Form, the Body–Cathexis Scale, the Rosenberg Self-Esteem Scale Short Form, and the Sexual Attitude Scale for Elderly People were used for data collection. For the data analysis, Pearson’s *r* correlation test, simultaneous multiple linear regression, and binary logistic regression were employed. The mean age of the participants was 69.97 ± 5.74 years, and 66.9% were between 65 and 74 years of age. Furthermore, 53.8% of the participants were male and 56.2% had two chronic illnesses. It was found that the participants’ age, body image, and self-esteem explained 84% of the total variance in their permissive attitudes toward sexuality. Furthermore, as the age of the older adults with chronic illnesses increased, there were negative effects on their permissive attitudes toward sexuality. However, as their body image and self-esteem levels improved, there was a positive effect on their permissive attitudes toward sexuality. These findings will be of benefit in encouraging and developing sexual health in older adults with chronic illnesses.

## Introduction

Sexuality is experienced and expressed in people’s thoughts, fantasies, desires, beliefs, attitudes, values, behaviors, practices, roles, and relationships. With regard to sexuality and relationship needs, people over the age of 50 have been commonly—and certainly historically—perceived as distant from sexuality and essentially invisible (World Health Organization, 2023). In the normal aging process, the change from middle age (< 65 years of age) to older age (≥ 65) leads to physical changes in both men and women (Finkenzeller et al., 2019). Normal physiological changes in older age, such as vaginal atrophy, reduction in vaginal secretions, and vaginal epithelial smoothing in women as well as erectile dysfunction in men, have negative effects on sexual life and pleasure. In addition, the possibility of urinary tract or bladder infections and the fear of urinary incontinence often cause women to avoid sexual activity (Chang et al., 2019; Reyhan et al., 2018). Other factors affecting sexuality in older adults include weight and body changes, hair loss, menopause, night sweats, body odor, chronic illnesses, and continuous medication use (Kvalem et al., 2020; Olchowska-Kotala, 2018; Sánchez-Cabrero et al., 2019; Séjourné et al., 2019).

Approximately 95% of older adults have at least one chronic illness, and approximately 80% have two or more chronic illnesses (Centers for Disease Control and Prevention, 2022). Some drugs used by older adults with more than one chronic illness may negatively affect the quality of their sexual lives (Aguiar et al., 2020). The increasing incidence of chronic illnesses in conjunction with an increase in the world population of older adults has engendered concern over the effects on sexual health. Chronic illnesses may have negative effects on sexual appetite, sexual function, body image, energy levels, self-esteem, interpersonal interaction, and quality of life (Merghati-Khoei et al., 2016; Træen & Villar, 2020).

Self-esteem can be defined as “taking oneself on, trusting and respecting oneself, and the individual seeing and approving of him/herself as valuable” (Olchowska-Kotala, 2018; Yılmaz, 2018). Body image has been defined as a person’s “perceptions, thoughts, and feelings regarding their body” and as their behavior regarding their body (Rica et al., 2018; Shkolnik & Iecovich, 2013; Thorpe et al., 2015; Træen & Villar, 2020). There are two main factors associated with this: (1) a perceptual dimension involving a person’s judgments about their own body size and proportions and (2) a cognitive–emotional dimension known as general body satisfaction (Sánchez-Cabrero et al., 2019). In essence, how people see their bodies affects what they feel about themselves. A person’s negative perceptions about their body cause low self-esteem (Chang et al., 2019; Sánchez-Cabrero et al., 2019). People with low self-esteem exhibit low satisfaction with life, low self-confidence, loneliness, depression, anxiety, and worry (Cybulski et al., 2018; Šare et al., 2021; Watt & Konnert, 2020; Yılmaz, 2018).

Studies on the sexuality of older adults are limited (Çengelci Özekes et al., 2022). In the literature (Olchowska-Kotala, 2018; Towler et al., 2021; Træen & Villar, 2020), it is emphasized that there is a need for studies with a wider sample group to determine how body image and self-respect in older adults can benefit the quality of their sexual health. However, the extent to which body image plays a role in sexual satisfaction remains ambiguous (Kvalem et al., 2020). Chronic illnesses can significantly affect older adults’ experiences of sexual life. Gaining a more in-depth understanding of the views and needs related to the sexual lives of older adults with chronic illnesses can provide an opportunity to shape permissive attitudes toward sexuality among them, as insufficient or incorrect knowledge about sexuality leads to the development of prejudiced behavior and conservative attitudes in older adults (Aguiar et al., 2020).

### Current Study

Knowledge about sexuality can help identify risk and protective factors for older adults’ sexual and/or relationship concerns, and further supporting these areas may enhance quality of life and health among older adults with chronic illnesses (Bach et al., 2013; Diamond & Huebner, 2012; Miguel et al., 2025). The primary aim of the current study was to determine the effect of older adults’ body image and self-esteem levels on their attitudes toward sexuality. The second aim was to examine the effect of the association between body image and self-esteem levels on attitudes toward sexuality in older adults with chronic illnesses. The following research questions were designed to realize these aims:

- What are the body image levels of older adults with chronic illnesses?
- What are the self-esteem levels of older adults with chronic illnesses?
- What are the levels of permissive attitudes toward sexuality of older adults with chronic illnesses?
- What is the correlation between older adults’ age, body image, and self-esteem and their permissive attitudes toward sexuality?
- What is the effect ratio between older adults’ age, body image, and self-esteem and their permissive attitudes toward sexuality?

## Method

### Participants

This research was descriptive, cross-sectional, and correlational. The target population was older adults aged 65 and above who were visiting family health centers in a province in the east of Türkiye. On average, 100 older adults visit these health centers each day.

The research sample comprised 1,004 older adults (*n* = 1004). A post hoc power analysis was conducted with the software program G*Power 3.1.9.7. For calculating the power, the sample size was set as 1,004; the confidence interval was set as 95%; and the significance level was set as *p* = 0.05. The power of the study was determined to be *0.99*, and the effect size was at a medium level (*0.25*). It was concluded that the sample number represented high power.

The sample group was selected using quota sampling, which is a nonprobability sampling method. In this method, only samples possessing certain characteristics that reflect the population being studied are selected (Etikan & Bala, 2017). Of the 1,004 participants, 463 (46.2%) were female and 541 (53.8%) were male. Their mean age was 69.97 ± 5.74 years, and 66.9% were in the 65–74 age range. Furthermore, 612 (60.9%) of the participants were married, 294 (29.3%) had primary-level education, 702 (70.0%) were not working, and 421 (41.9%) had incomes equal to their expenditures. Table 1 shows the other demographic characteristics of the participants.

**Table 1:** Demographic characteristics of older adults with chronic illnesses (*n* = 1004)

Variables Mean ± SD	n (%)
*Body mass index (BMI) (kg/m*^*2*^*) 20.17 ± 3.59*	
18.5 and below (weak)	463 (46.1)
18.6–24.9 (normal weight)	348 (34.6)
25.0 and above (overweight)	193 (19.3)
*Number of chronic diseases*	
One	254 (25.3)
Two	565 (56.2)
Three or more	185 (18.5)
Continuously used drugs*	
Antihypertensive	207 (20.7)
Antidiabetic	203 (20.3)
Cardiovascular drugs	119 (11.9)
Bronchodilator	112 (11.1)
Stomach medications	105 (10.4)
Anti-Inflammatory analgesic	103 (10.2)
Antirheumatic	83 (8.3)
Psychotropic sedatives and hypnotics drugs	71 (7.1)
*Active sex life*	
Yes	601 (59.9)
No	403 (40.1)
Frequency of sexual intercourse**	
Once a week	155 (25.7)
2–3 times weekly	123 (20.4)
Once 2 weeks	172 (28.6)
Once per month	101 (16.9)
A few times a year	50 (8.4)

*Participants were allowed to give more than one response, **percentages are based on n = *601*

The inclusion criteria were individuals who were aged 65 years or more, who attended any of the family health centers, who had at least one chronic illness, who were able to communicate, and who agreed to participate in the research.

The exclusion criteria were individuals who were below the age of 65; did not have any chronic illnesses; had cognitive dysfunction; had mental illness diagnoses; had psychiatric illnesses requiring medical intervention; wished to withdraw from the research at any stage of the study; filled out the data collection forms wrongly or incompletely; left questions half-answered or did not want to answer the questions; or did not give their approval on the informed consent form.

Five people were removed from the research because they filled out the forms incorrectly or incompletely, and 12 who did not give their approval on the informed consent form were not included. Furthermore, 101 participants who did not have any chronic illnesses were excluded, and 12 who found the questions shameful withdrew at some stage of the study.

## Measures

### Older Person’s Description Form

In line with the literature (Ebeoğlu et al., 2021; Kvalem et al., 2020; Šare et al., 2021; Séjourné et al., 2019), this form was created by the researchers to gather data on age, body mass index (BMI) (kg/m^2^), sex, marital status, education level, working status, monthly income, number of chronic illnesses, continuously used drugs, active sex life, and frequency of sexual intercourse. The form consisted of 11 questions.

BMI was calculated according to the weight (kg) and height (m) stated by each participant, using the formula BMI = kg/m^2^.

### Body-Cathexis Scale

The BCS was created by Secord and Jourard (1953) and adapted for Turkish individuals by Hovardaoğlu (1992). This self-reporting form was devised to measure a person’s level of discontent with parts of their body or bodily functions. It is a 5-point Likert-type scale that consists of 40 items. The items are scored between 1 and 5; the most positive statement scores 1 point, whereas the most negative one scores 5 points, with the choices comprising “I like it very much,” “I quite like it,” “I am undecided,” “I don’t much like it,” and “I don’t like it at all.” The lowest possible score is 40, and the highest is 200. A higher total score obtained on the scale shows less satisfaction with parts or functions of the body, whereas a lower total score indicates greater satisfaction (Hovardaoğlu, 1992).

The Cronbach’s alpha internal consistency coefficient of the original scale was 0.91 (Hovardaoğlu, 1992), and in the current study, it was 0.97.

### Short Form Rosenberg Self-Esteem Scale

The SF-RSES was developed by Rosenberg (1965) and adapted for Turkish individuals by Çuhadaroğlu (1986). It consists of 63 items and 12 subdimensions. In line with the aim of the current research, the first 10 items of the scale were used to measure self-esteem (Çuhadaroğlu, 1986). Of these 10 items, five are positive and five negative. The scale is a 4-point Likert-type one, with 6 as the highest possible score and 0 as the lowest. The scale results can be categorized into three groups: those who score 0–1 have high self-esteem; those who score 2–4 have moderate self-esteem; and those who score 5–6 have low self-esteem. Thus, a low score on the scale indicates high self-esteem, whereas a high score indicates low self-esteem (Çuhadaroğlu, 1986).

The Cronbach’s alpha internal consistency coefficient for the Turkish version was 0.77 (Çuhadaroğlu, 1986), and in the current research, it was 0.79.

### The Sexual Attitude Scale for Elderly People

The Sexual Attitude Scale for Elderly People was devised by Park et al. (2014) and adapted to the Turkish language by Ebeoğlu et al. (2021). It has 18 items and four subdimensions. The subdimensions are volunteerism (Items 1–6), importance (Items 7–10), closeness (Items 11–15), and communication (Items 16–18). Furthermore, it is a 5-point Likert scale, and the items are scored from 1 (“I definitely disagree”) to 5 (“I definitely agree”). There are no reverse-scored items on the scale. The minimum possible score is 18, and the maximum is 90. High scores show that older adults have a permissive or positive attitude toward older-age sexuality (Ebeoğlu et al., 2021).

The Cronbach’s alpha internal consistency coefficient of the original scale was 0.93 (Ebeoğlu et al., 2021), and in the current research, it was 0.95.

### Procedure

The data collection took place between December 2022 and August 2023. A face-to-face interview technique was used, and the questionnaire was completed in a calm, quiet room. If the older person was unable to complete the form (e.g., because of eyesight problems or illiteracy), the researcher read the questions to the older person and completed the questionnaire according to the answers given. The data collection took approximately 20–25 min.

### Statistical Analysis

Data analysis was carried out using the Statistical Package for Social Sciences (SPSS) program for Windows (Version 25.0). Conformity of the data to normal distribution was analyzed using the Shapiro–Wilk test and Q–Q graphic examinations. A post hoc power analysis was conducted with the software program G*Power 3.1.9.7. Continuous variables such as age and BMI were given as the mean ± standard deviation, and categorical variables were presented as numbers and percentages. In the analysis of data showing a normal distribution, Pearson’s *r* correlation test, simultaneous multiple linear regression, and binary logistic regression were used. Pearson’s *r* correlation test was employed to examine the relationships between variables. Simultaneous multiple linear regression analysis was performed to examine whether age, body image levels, and self-esteem levels could explain permissive attitudes toward sexuality in older adults with chronic illnesses. Binary logistic regression analysis was performed to determine the demographic characteristic variables predicting the effect of attitudes toward sexuality in older adults with chronic illnesses. The effect sizes of the criteria used for the correlations were as follows: 0.10–0.30, small; 0.30–0.50, moderate; and > 0.50, large (Cohen, 1988). Statistical significance was taken as *p* < 0.05.

## Results

Table 2 presents the descriptive statistics (mean and standard deviation) and correlations between measures (Pearson’s *r*). Table 3 shows the results of the multiple linear regression analysis performed to determine the effects relating to the mean scores of the Sexual Attitude Scale for Elderly People (the entire scale as well as the subdimensions of volunteerism, significance, closeness, and communication), age, the BCS, and SF-RSES. The overall model was found to be significant. Based on this, the older adults’ age, body image, and self-esteem explained 84% of the total variance in their attitudes toward sexuality. In the regression model, when the results of the *t* test on the significance of the regression coefficient were examined, an increase in the age of the participants statistically reduced their permissive attitudes toward sexuality. However, an increase in the levels of the volunteerism subdimension score, significance subdimension score, closeness subdimension score, communication subdimension score, BCS total score, and SF-RSES total score might statistically increase permissive attitudes toward sexuality.

**Table 2:** Descriptive statistics of scale scores and correlations between measurements (Pearson’s *r*)

Variables		1		2		3		4		5		6		7		8	
	Mean** ± ***SD*	*r*	*p*	*r*	*p*	*r*	*p*	*r*	*p*	*r*	*p*	*r*	*p*	*r*	*p*	*r*	*p*
Age (1)		–	–	−0.033	.379	–0.301	.007	–0.059	.118	−0.038	.319	−0.366	.048	−0.100	.008	−0.312	.003
Volunteerism (2)	15.92 ± 6.36	−0.033	.379	–	–	0.702	*p* < .001	0.604	*p* < .001	0.504	*p* < .001	0.834	*p* < .001	0.397	*p* < .001	0.284	*p* < .001
Significance (3)	11.84 ± 5.24	−0.301	.007	0.702	*p* < .001	–	–	0.783	*p* < .001	0.636	*p* < .001	0.903	*p* < .001	0.454	.004	0.211	*p* < .001
Closeness (4)	16.43 ± 6.10	−0.059	.118	0.604	*p* < .001	0.783	*p* < .001	–	–	0.775	*p* < .001	0.910	*p* < .001	0.411	.007	0.177	*p* < .001
Communication (5)	10.48 ± 4.13	−0.038	.319	0.504	*p* < .001	0.636	*p* < .001	0.775	*p* < .001	–	–	0.812	*p* < .001	0.450	.015	0.094	.013
The Sexual Attitude Scale for Elderly People Total (6)	54.68 ± 18.95	−0.366	.048	0.834	*p* < .001	0.903	*p* < .001	0.910	*p* < .001	0.812	*p* < .001	–	–	0.873	*p* < .001	0.431	*p* < .001
BCS Total (7)	108.64 ± 38.31	0.100	.008	0.397	*p* < .001	0.454	.004	0.411	.007	0.450	.015	0.873	*p* < .001	–	–	0.442	*p* < .001
SF-RSES Total (8)	2.00 ± 0.49	−0.312	.003	0.284	*p* < .001	0.211	*p* < .001	0.177	*p* < .001	0.094	.013	0.431	*p* < .001	0.442	*p* < .001	–	–

^*^ SD: standard deviation, ^**^ BCS, Body–Cathexis Scale; SF-RSES, Rosenberg Self-Esteem Scale Short Form

**Table 3:** Simultaneous multiple linear regression analysis of attitudes toward sexuality in older adults with chronic illnesses

	The Sexual Attitude Scale for Elderly People total				
Model	B	Standardized coefficients β	*t*	*p*	VIF
(Constant)	81.66		9.30	*p* < .001	
Age	−9.30	.000	−5.92	*p* < .001	1.21
Volunteerism	3.33	.001	7.28	*p* < .001	1.15
Significance	3.27	.001	6.11	*p* < .001	1.30
Closeness	3.32	.001	7.45	*p* < .001	1.55
Communication	3.21	.001	5.04	*p* < .001	1.29
BCS total	2.51	.000	39.39	*p* < .001	1.32
SF-RSES total	9.44	.000	6.03	*p* < .001	1.25

* R = 0.84, R2 = 0.70, F = 20.19, *p* < .001, Durbin–Watson = 1.90. ** BCS,  Body–Cathexis Scale; SF-RSES, Rosenberg Self-Esteem Scale Short Form

It was found that being between the ages of 74 and 85; being female; being single, divorced, or widowed; being illiterate; having two chronic illnesses; using cardiovascular drugs; using psychotropic sedatives and hypnotic drugs; not having an active sex life; and having sexual relations once in two weeks were risk factors affecting the older adults’ permissive attitudes toward sexuality (Table 4).

**Table 4:** Regression analysis for demographic characteristic variables predicting the effect of attitudes toward sexuality in older adults with chronic illnesses

	Univariate			
Variables	Β	*p*	OR	95% CI
Being in the 74–85 age range	−.016	.021	1.01	1.00–1.03
Being female	−.027	.003	0.97	0.95–0.99
Being single, divorced, or widowed	−.011	.020	0.98	0.98–0.99
Having two chronic illnesses	−.041	.042	1.04	1.00–1.08
Using cardiovascular drugs	−.013	.045	1.01	1.00–1.02
Using psychotropic sedatives and hypnotic drugs	−.119	.025	0.80	0.72–0.93
Not having an active sex life	−.029	.036	0.97	0.94–0.99

OR = odds ratio

## Discussion

Sexuality is an important part of life for older adults with chronic illnesses. In a systematic review of 98 articles, Merghati-Khoei et al. (2016) found that chronic illnesses negatively affected older adults’ sexual lives, sexual functions, sexual performance, and close relationships. In the current study, it was found that the sexual attitudes of the older adults with chronic illnesses were negatively affected by their age and predicted by their levels of body image perception and self-esteem. The regression analysis revealed that the age, body image, and self-esteem of the older adults explained 84% of the total variance in their sexual attitudes. In particular, age, body image, and self-esteem emerged as the strongest predictors.

It was also found that, as body image perception and self-esteem levels improved, there was a positive effect on permissive attitudes toward sexuality (Table 3). Thus, the findings support body image perception and self-esteem levels playing a positive role and being positive predictors of older adults’ attitudes toward sexuality. The findings also indicate that age plays a negative role and is a negative predictor of older adults’ attitudes toward sexuality. Séjourné et al. (2019) conducted qualitative research with 357 women entering menopause, finding that their dissatisfaction with their body image was significantly high and that their sexual dissatisfaction significantly increased. Despite the limitations mentioned below, the current study has confirmed the correlation with higher self-esteem, thereby advancing the understanding of the sexual health of older adults. Kvalem et al. (2020), Shkolnik and Iecovich (2013), and Vasconcelos et al. (2022) found a weak correlation between body image and sexual satisfaction in older adults. In addition, it has been found that a negative self-image is widespread among individuals with sexual discontent (Chang et al., 2019).

In the current study, as the age of the older adults increased, a greater effect was seen on permissive attitudes toward sexuality (Table 3). In other words, as the age of the older adults with chronic illnesses increased, their permissive attitudes toward sexuality decreased. This is in line with the research of Çengelci Özekes et al. (2022), who found a weak, statistically significant negative correlation between age and attitudes toward older-age sexuality. With advancing age, Turkish participants may hold mistaken beliefs that sexuality is not socially acceptable, will increase health problems, and is not appropriate from a religious perspective. However, the permissive attitudes toward sexuality of people living in other societies may vary. Therefore, similar studies should be conducted in different societies and cultures.

In the current study, a negative correlation at a moderate level of significance was found between age and permissive attitudes toward sexuality in the older adults with chronic illnesses. Furthermore, a strongly significant positive correlation was found between the older adults’ body image perceptions and their attitudes toward sexuality (Table 2). This result may indicate that, as the body image perceptions of older adults with chronic illnesses improve, their permissive attitudes toward older-age sexuality increase. In addition, a positive correlation at a moderate level of significance was found between the older adults’ self-esteem and their attitudes toward sexuality (Table 2). This result may indicate that having negative self-esteem during sexual activity distances older adults from a positive attitude toward sexuality.

In this study, a positive correlation at a moderate level of significance was found between the older adults’ body image perceptions and their self-esteem (Table 2). Bergman (2021) conducted a study with 383 older adults and found a moderate-level positive correlation between self-esteem and body image (*r* = 0.44, *p* < 0.001). In Séjourné et al.’s (2019) quantitative research involving 357 women entering menopause, a moderate level of negative correlation was found between body satisfaction and self-esteem. Furthermore, Olchowska-Kotala’s (2018) study with 68 older adults showed a strong level of positive correlation between self-esteem and body image. In a study by Watt and Konnert (2020) with 150 older adults, no significant correlation was found between self-esteem and body image; however, a negative correlation at a moderate level was found between age and self-esteem.

In the current research, the following characteristics were determined to be risk factors affecting the older adults’ attitudes toward sexuality: being between the ages of 74 and 85; being female; being single, divorced, or widowed; being illiterate; having two chronic illnesses; using cardiovascular drugs; using psychotropic sedatives and hypnotic drugs; not having an active sex life; and having sexual relations once a week (Table 4). Many drugs that are widely used to treat chronic illnesses have negative effects on sex drive and sexual function. Hypertension, coronary heart disease, and congestive heart failure may be correlated with increased sexual dysfunction. When older patients are given sexual counseling, they must be asked about the drugs that they are taking regularly. It is known that drugs such as beta blockers and diuretics cause a loss of libido as well as erectile and ejaculation dysfunction. Furthermore, the iatrogenic effects of drugs, including antidepressive and antihypertension drugs, beta blockers, and drugs given for the side effects of chemotherapy and radiotherapy, may have adverse effects on one’s sex drive (Merghati-Khoei et al., 2016). In a study conducted in Israel on men and women over the age of 60 living with a spouse or partner, a correlation was reported between body satisfaction and sexual satisfaction. However, when control variables such as age, gender, education, and sexual activity were included, body image no longer predicted sexual satisfaction, particularly when they had serious illnesses such as cardiovascular diseases, diabetes, or depression (Shkolnik & Iecovich, 2013). This may indicate a need to reassess the role and importance of sexuality at this stage in people’s lives.

A BCS score near 200 indicates a person’s dissatisfaction with the areas or functions of the body. In the current study, the older adults’ body image perception scores were found to be at a moderate level (Table 2). Hence, it can be said that the older adults were not content with their body image perceptions. Numerous studies have presented similar conclusions (Cameron et al., 2019; Kvalem et al., 2020; Olchowska-Kotala, 2018; Séjourné et al., 2019; Shkolnik & Iecovich, 2013; Thorpe et al., 2015). However, studies have also found (Menezes et al., 2014; Rica et al., 2018; Sánchez-Cabrero et al., 2019) older adults to be content with their body perceptions. The low body perception satisfaction levels of the older adults in the current study might have resulted from their BMI scores, as 46.1% of the participants had a BMI of 18.5 or below (Table 1).

In this study, the SF-RSES total mean score was found to be 2.00 ± 0.49 (Table 2). According to the interpretation of the scale, those participants who scored 2–4 had moderate self-esteem. Thus, the self-esteem of the participants was at a moderate level. In Rosenberg’s approach, high general self-esteem is formed by a person feeling that they are “good enough” and a valuable person, whereas low self-esteem manifests as a lack of satisfaction and discontent with the self (Olchowska-Kotala, 2018). Other studies in the literature have found similar results (Chang et al., 2019; Olchowska-Kotala, 2018; Šare et al., 2021; Séjourné et al., 2019; Watt & Konnert, 2020; Yılmaz, 2018). In contrast, Szcześniak et al. (2020) found a high level of self-esteem in 179 women; this difference may be because their sample population (*n* = 179) was small and a majority (65%) of their participants were female.

It is generally accepted that, when the score obtained on the Sexual Attitude Scale for Elderly People approaches 90, the person’s attitude toward sexuality is permissive or positive. In this research, the permissive attitudes toward sexuality of the older adults with chronic illnesses were at a moderate level (Table 2). An aspect that distinguishes the current research from other studies is that only older adults with chronic illnesses were selected as the sample group. Using a scale similar to the one in the current research, Çengelci Özekes et al. (2022) found that the permissive attitudes toward sexuality of 127 older adults were low. In other studies that used a different scale—the Aging Sexual Knowledge and Attitudes Scale—it was found that older adults’ attitudes toward sexuality were at a moderate level (Aguiar et al., 2020; Cybulski et al., 2018; Kiziltepe et al., 2022). Furthermore, upon conducting qualitative interviews on sexuality with 31 older adults in the 66–92 age range, Towler et al. (2021) found that this group viewed sex as “past it” and “irrelevant.” Overall, the conclusions of the current study conform to the existing literature.

### Limitations

First, this study used quota sampling and had a cross-sectional design. As only volunteer participants were involved in the study, there was selection bias. Second, questions regarding sexuality are generally viewed as a sensitive topic and may cause shame or shyness in some participants in older age groups. This may lead to exaggerated or censored answers to questions concerning sexual experiences. Third, an analysis of the BMI levels of the older adults involved in this research was not included. This is because progressive shrinking of the spine in older adults may cause BMI scores to appear greater or less, leading to incorrect interpretations. Finally, the study sample consisted solely of older adults with chronic illnesses who were visiting family health centers in a province in the east of Türkiye. Therefore, the research conclusions are applicable only to the patients to whom the questionnaire was given and cannot be generalized to other populations.

### Conclusion

A positive correlation at a strong level of significance was found between the body image perceptions and attitudes toward sexuality of the older adults with chronic illnesses who participated in this study. Furthermore, a positive correlation at a medium level of significance was found between the older adults’ self-esteem and their attitudes toward sexuality, and a positive correlation at a strong level of significance was found between body image perception and self-esteem. The older adults’ age, body image, and self-esteem explained 84% of the total variance in attitudes toward sexuality. Moreover, greater age was found to have negative effects on the older adults’ permissive attitudes toward sexuality. However, it was also found that, as body image and self-esteem levels improved, there was a positive effect on attitudes toward sexuality.

Being between the ages of 74 and 85; being female; being single, divorced, or widowed; being illiterate; having two chronic illnesses; using cardiovascular drugs; using psychotropic sedatives and hypnotic drugs; not having an active sex life; and having sexual relations once in two weeks were risk factors affecting the older adults’ permissive attitudes toward sexuality.

Overall, this research found that the older adults with chronic illnesses had negative body image perceptions and moderate levels of self-esteem. Furthermore, they had permissive attitudes toward sexuality at a moderate level. Examining the participants’ scores on the subdimensions of the Sexual Attitude Scale for Elderly People showed that, in general, their volunteerism, significance, closeness, and communication scores were at a moderate level.

These findings can aid in encouraging and developing sexual health in older adults with chronic illnesses. The findings, unlike those of several studies in the literature, provide information on both male and female participants. Thus, the findings can be important in gaining further insights on sexual attitudes, body image, and self-esteem levels in individuals with and without chronic illnesses in a clinical setting. There is a need for public health programs to improve attitudes toward sexual health in older adults with chronic illnesses and to enhance their body image and self-esteem. Intervention programs aimed at improving body image must be arranged for older adults with chronic illnesses.

Few existing qualitative studies have addressed the correlation between the body image, self-esteem, and attitudes toward sexuality of older adults with chronic illnesses. Therefore, it is recommended that studies utilizing qualitative research methods be conducted in the future to assess the views and actions of older adults with chronic illnesses regarding their attitudes toward sexuality.

## References

[CR1] Aguiar, R. B., Leal, M. C. C., de Marques, A. P., & O. (2020). Knowledge and attitudes about sexuality in the elderly with hiv. *Ciência & Saúde Coletiva,**25*(6), 2051–2062. 10.1590/1413-81232020256.18432018 32520253 10.1590/1413-81232020256.18432018

[CR2] Bach, L. E., Mortimer, J. A., VandeWeerd, C., & Corvin, J. (2013). The association of physical and mental health with sexual activity in older adults in a retirement community. *Journal of Sexual Medicine,**10*(11), 2671–2678. 10.1111/jsm.12308 23981252 10.1111/jsm.12308

[CR3] Bergman, Y. S. (2021). Ageism and psychological distress in older adults: The moderating role of self-esteem and body image. *Journal of Applied Gerontology,**41*(3), 836–841. 10.1177/07334648211009658 33913366 10.1177/07334648211009658

[CR4] Cameron, E., Ward, P., Mandville-Anstey, S. A., & Coombs, A. (2019). The female aging body: A systematic review of female perspectives on aging, health, and body image. *Journal of Women and Aging,**31*(1), 3–17. 10.1080/08952841.2018.1449586 29558298 10.1080/08952841.2018.1449586

[CR5] Çengelci Özekes, B., Ebeoğlu Duman, M., & Kızıltepe, R. (2022). An investigation of factors related to attitudes towards elderly sexuality in older adulthood: A cross-sectional study. *Turkiye Klinikleri Journal of Health Sciences,**7*(4), 1112–1119. 10.5336/healthsci.2022-89494

[CR6] Centers for Disease Control and Prevention. (2023, May). *About chronic diseases*. Retrieved from: https://www.cdc.gov/chronicdisease/about/index.htm

[CR7] Chang, S.-R., Yang, C. F., & Chen, K.-H. (2019). Relationships between body image, sexual dysfunction, and health-related quality of life among middle-aged women: A cross-sectional study. *Maturitas,**126*, 45–50. 31239117 10.1016/j.maturitas.2019.04.218

[CR8] Cohen, J. (1988). *Statistical power analysis for the behavioral sciences*. Lawrence Erlbaum Associates.

[CR9] Çuhadaroğlu, F. (1986) *Self-esteem in adolescents* (Unpublished master's thesis). Retrieved from: https://tez.yok.gov.tr/UlusalTezMerkezi/

[CR10] Cybulski, M., Cybulski, L., Krajewska-Kulak, E., Orzechowska, M., Cwalina, U., & Jasinski, M. (2018). *Sexual quality of life, sexual knowledge, and attitudes of older adults on the example of inhabitants over 60s of Bialystok, Poland*. *Frontiers in Psychology*, *9*. 10.3389/fpsyg.2018.00483 10.3389/fpsyg.2018.00483 PMC5904191 29695983

[CR11] de Menezes, T. N., Brito, K. Q. D., Oliveira, E. C. T., & Pedraza, D. F. (2014). Body image perception and associated factors among elderly residents in a city in northeast Brazil: A population-based study. *Ciencia & Saude Coletiva,**19*(8), 3451–3460. 10.1590/1413-81232014198.15072013 25119084 10.1590/1413-81232014198.15072013

[CR12] Diamond, L. M., & Huebner, D. M. (2012). Is good sex good for you? Rethinking sexuality and health. *Social and Personality Psychology Compass,**6*(1), 54–69.

[CR13] Ebeoğlu, M., Kiziltepe, R., & Çengelci Özekes, N. B. (2021). Sexual attitude scale for elderly people: A reliability and validity study in turkey. *Turkish Journal of Geriatrics,**24*(3), 433–440.

[CR14] Etikan, I., & Bala, K. (2017). Sampling and sampling methods. *Biometrics & Biostatistics International Journal,**5*(6), 5–7.

[CR15] Finkenzeller, T., Pötzelsberger, B., Kösters, A., Würth, S., Amesberger, G., Dela, F., & Müller, E. (2019). Aging in high functioning elderly persons: Study design and analyses of behavioral and psychological factors. *Scandinavian Journal of Medicine & Science in Sports*, *29*, 7–16. 10.1111/sms.13368 30570174 10.1111/sms.13368 PMC6850373

[CR16] Hovardaoğlu, S. (1992). Body-Cathexis Scale. *Journal of Psychiatry Psychology Psychopharmacology (3P), 1*(1), 11–26. (in Turkish).

[CR17] Kiziltepe, R., Ebeoğlu Duman, M., & Çengelci Özekes, N. B. (2022). Knowledge and attitudes toward elderly sexuality: A comparison of young and older adults. *Turkish Journal of Geriatrics,**25*(1), 79–87.

[CR18] Kvalem, I. L., Graham, C. A., Hald, G. M., Carvalheira, A. A., Janssen, E., & Štulhofer, A. (2020). The role of body image in sexual satisfaction among partnered older adults: A population-based study in four European countries. *European Journal of Ageing,**17*(2), 163–173. 10.1007/s10433-019-00542-w 32549871 10.1007/s10433-019-00542-w PMC7292835

[CR19] Merghati-Khoei, E., Pirak, A., Yazdkhasti, M., & Rezasoltani, P. (2016). Sexuality and elderly with chronic diseases: A review of the existing literature. *Journal of Research in Medical Sciences,**21*, 136. 10.4103/1735-1995.196618 28331522 10.4103/1735-1995.196618 PMC5348839

[CR20] Miguel, I., von Humboldt, S., & Leal, I. (2025). Sexual well-being across the lifespan: Is sexual satisfaction related to adjustment to aging? *Sexuality Research and Social Policy*, *22*, 306–317. 10.1007/s13178-024-00939-y

[CR21] Olchowska-Kotala, A. (2018). Body esteem and self-esteem in middle-aged women. *Journal of Women & Aging,**30*(5), 417–427. 28453407 10.1080/08952841.2017.1313012

[CR22] Park, H., Shin, S., & Cha, H. (2014). Development and validation of a sexual attitude scale for elderly korean people. *Journal of Korean Gerontological Nursing,**16*(3), 189–200.

[CR23] Reyhan, F., Özerdoğan, N., & Arık, E. (2018). A neglected subject: Sexualıty in elderly. *Journal of Health Sciences, 27*(1), 76–79. https://dergipark.org.tr/tr/pub/eujhs/issue/44573/553218

[CR24] Rica, R. L., Gama, E. F., Machado, A. F., Alonso, A. C., Evangelista, A. L., Figueira-Junior, A., Zanetti, M., Brandão, R., de Jesus Miranda, M. L., Alves, J. V., Bergamin, M., & Bocalini, D. S. (2018). Does resistance training improve body image satisfaction among the elderly? A cross-sectional study. *Clinics*, *73*. 10.6061/clinics/2018/e290 10.6061/clinics/2018/e290 PMC6038056 30088536

[CR25] Sánchez-Cabrero, R., León-Mejía, A. C., Arigita-García, A., & Maganto-Mateo, C. (2019). Improvement of body satisfaction in older people: An experimental study. *Frontiers in Psychology,**10*. 10.3389/fpsyg.2019.02823 10.3389/fpsyg.2019.02823 PMC6920176 31920858

[CR26] Šare, S., Ljubičić, M., Gusar, I., Čanović, S., & Konjevoda, S. (2021). Self-esteem, anxiety, and depression in older people in nursing homes. *Healthcare*, *9*, 1035. 10.3390/healthcare9081035 34442172 10.3390/healthcare9081035 PMC8392518

[CR27] Secord, P. F., & Jourard, S. M. (1953). The appraisal of body-cathexis: Body-cathexis and the self. *Journal of Consulting Psychology,**17*(5), 343–347. 10.1037/h0060689 13109086 10.1037/h0060689

[CR28] Séjourné, N., Got, F., Solans, C., & Raynal, P. (2019). Body image, satisfaction with sexual life, self-esteem, and anxiodepressive symptoms: A comparative study between premenopausal, perimenopausal, and postmenopausal women. *Journal of Women & Aging,**31*(1), 18–29. 10.1080/08952841.2018.1510247 30152729 10.1080/08952841.2018.1510247

[CR29] Shkolnik, D., & Iecovich, E. (2013). Health, body image, gender, and migration status: Their relationship to sexuality in old age. *International Psychogeriatrics,**25*(10), 1717–1727. 10.1017/S1041610213000604 23642354 10.1017/S1041610213000604

[CR30] Szcześniak, M., Bielecka, G., Madej, D., Pieńkowska, E., & Rodzeń, W. (2020). The role of self-esteem in the relationship between loneliness and life satisfaction in late adulthood: Evidence from poland. *Psychology Research and Behavior Management,**13*, 1201–1212. 10.2147/PRBM.S275902 33363419 10.2147/PRBM.S275902 PMC7754268

[CR31] Thorpe, R., Fileborn, B., Hawkes, G., Pitts, M., & Minichiello, V. (2015). Old and desirable: Older women’s accounts of ageing bodies in intimate relationships. *Sexual and Relationship Therapy,**30*(1), 156–166. 10.1080/14681994.2014.959307 10.1080/14681994.2014.936722 PMC4270421 25544829

[CR32] Towler, L. B., Graham, C. A., Bishop, F. L., & Hinchliff, S. (2021). Older adults’ embodied experiences of aging and their perceptions of societal stigmas toward sexuality in later life. *Social Science & Medicine,**287*, 114355. 34474307 10.1016/j.socscimed.2021.114355

[CR33] Træen, B., & Villar, F. (2020). Sexual well-being is part of aging well. *European Journal of Ageing,**17*(2), 135–138. 10.1007/s10433-020-00551-0 32549868 10.1007/s10433-020-00551-0 PMC7292839

[CR34] Vasconcelos, P., Paúl, C., Serruya, S. J., de León, R. G. P., & Nobre, P. (2022). A systematic review of sexual health and subjective well-being in older age groups. *Pan American Journal of Public Health,**46*. 10.26633/RPSP.2022.179 10.26633/RPSP.2022.179 PMC9595221 36320206

[CR35] Watt, A. D., & Konnert, C. A. (2020). Body satisfaction and self-esteem among middle-aged and older women: The mediating roles of social and temporal comparisons and self-objectification. *Aging and Mental Health,**24*(5), 797–804. 10.1080/13607863.2018.1544222 30588850 10.1080/13607863.2018.1544222

[CR36] World Health Organization (WHO). (2023, May). *Sexual and reproductive health and research (SRH)*. Retrieved from: https://www.who.int/teams/sexual-and-reproductive-health-and-research/key-areas-of-work/sexual-health/defining-sexual-health

[CR37] Yılmaz, E. (2018). Relation of depression and self-esteem in older nursing homes. *Kalem İnternational Journal of Education and Human Sciences,**8*(2), 553–578.

